# Interaction of calcium responsive proteins and transcriptional factors with the PHO regulon in yeasts and fungi

**DOI:** 10.3389/fcell.2023.1225774

**Published:** 2023-08-03

**Authors:** Juan F. Martín

**Affiliations:** Departamento de Biología Molecular, Área de Microbiología, Universidad de León, León, Spain

**Keywords:** filamentous fungi, secondary metabolites biosynthesis, phosphate control, Pho4 regulator, inositol pyrophosphates, calcium homeostasis, Crz1(CrzA) transcriptional factor, acidocalcisomes

## Abstract

Phosphate and calcium ions are nutrients that play key roles in growth, differentiation and the production of bioactive secondary metabolites in filamentous fungi. Phosphate concentration regulates the biosynthesis of hundreds of fungal metabolites. The central mechanisms of phosphate transport and regulation, mediated by the master Pho4 transcriptional factor are known, but many aspects of the control of gene expression need further research. High ATP concentration in the cells leads to inositol pyrophosphate molecules formation, such as IP3 and IP7, that act as phosphorylation status reporters. Calcium ions are intracellular messengers in eukaryotic organisms and calcium homeostasis follows elaborated patterns in response to different nutritional and environmental factors, including cross-talking with phosphate concentrations. A large part of the intracellular calcium is stored in vacuoles and other organelles forming complexes with polyphosphate. The free cytosolic calcium concentration is maintained by transport from the external medium or by release from the store organelles through calcium permeable transient receptor potential (TRP) ion channels. Calcium ions, particularly the free cytosolic calcium levels, control the biosynthesis of fungal metabolites by two mechanisms, 1) direct interaction of calcium-bound calmodulin with antibiotic synthesizing enzymes, and 2) by the calmodulin-calcineurin signaling cascade. Control of very different secondary metabolites, including pathogenicity determinants, are mediated by calcium through the Crz1 factor. Several interactions between calcium homeostasis and phosphate have been demonstrated in the last decade: 1) The inositol pyrophosphate IP3 triggers the release of calcium ions from internal stores into the cytosol, 2) Expression of the high affinity phosphate transporter Pho89, a Na+/phosphate symporter, is controlled by Crz1. Also, mutants defective in the calcium permeable TRPCa7-like of *Saccharomyces cerevisiae* shown impaired expression of Pho89. This information suggests that CrzA and Pho89 play key roles in the interaction of phosphate and calcium regulatory pathways, 3) Finally, acidocalcisomes organelles have been found in mycorrhiza and in some melanin producing fungi that show similar characteristics as protozoa calcisomes. In these organelles there is a close interaction between orthophosphate, pyrophosphate and polyphosphate and calcium ions that are absorbed in the polyanionic polyphosphate matrix. These advances open new perspectives for the control of fungal metabolism.

## 1 Introduction

The ability to respond to multiple environmental conditions and different nutritional factors is critically important for the growth and differentiation of bacteria and fungi. This requires the integration of numerous sensing mechanisms and signaling cascades that respond to different inputs signals. Calcium is a well-known signaling molecule in the metabolism of filamentous fungi and many other eukaryotic cells. The calcium ions signaling process and its regulation has been studied in the yeast *Saccharomyces cerevisiae* but there is less information in filamentous fungi of the signaling mechanisms and their interaction with biotic and abiotic stress conditions (Martín and Liras, 2021a). Transient receptor potential (TRP) ion channels are non-selective calcium permeable cation transporters that have been very well studied in animal cells but the information in filamentous fungi is scarce (Venkatachalam and Montell, 2007; Martín and Liras, 2022b). The TRPs are related to members of the multiple facilitator superfamily (MFS) proteins which have 12 transmembrane domains, inserted in either plasma or organelle membranes, e.g. the Pho84 and Pho89 inorganic phosphate transporters of the multiple facilitator superfamily. In addition to their role in cation transport, TRP serve as cell sensors for physical and chemical signals such as osmolarity, high temperature and a variety of chemical substances (Clapham, 2003). Many canonical TRPs contain calmodulin binding domains (Zhu, 2005), EF-hand motives for calcium binding (Zurborg et al., 2007) and ankyrin repeats that appear to serve as a bite for interaction with a variety of stimuli. The presence of calmodulin-binding domains indicates that TRPs play an important role in the overall regulation of calcium homeostasis (see below). Several TRP channels have been identified in *S. cerevisiae*, *Candida albican*s and in some fungi, e.g. the TRP1 channel in *Giberella zeae* (Ihara et al., 2013) or those related to transport of intermediates or external stimuli in β-lactam antibiotic producing fungi such as Pen V in *Penicillium chrysogenum* and CefP in *Acremonium chrysogenum* (Martín and Liras, 2021b)*.* The *CaPhm7* gene*,* encoding a TRP in *C. albicans,* is a member of the calcium-permeable stress-gated cation transporters (CSC) and is involved in filamentation of this yeast, ion homeostasis, drug resistance and pathogenicity (Jiang and Pan, 2018). The homologous gene in *S. cerevisiae, ScPhm7*, has been found to be closely related with the PHO regulon members (Ogawa et al., 2000). Based on the available information it has been proposed that the *phm7* genes, both in *S. cerevisiae* and *C. albicans,* and the *penV* and *cefP* of *P. chrysogenum* and *A. chrysogenum*, respectively, play a role in calcium ion transport, connecting the effect of phosphate concentration with calcium homeostasis (see Section 3.3). This suggests that in yeasts there is an important connection between phosphate and calcium transport and signaling (Box 1). This hypothesis is supported by the finding that several genes of the PHO regulon are controlled by calcium through the calmodulin-calcineurin signaling cascade. Taking into account the previous knowledge this article is focused on the characterization of the interactions between calcium transport and signaling and phosphate regulation of fungal metabolism with special attention to the biosynthesis of bioactive secondary metabolites and the virulence of pathogenic fungi. First, the molecular mechanisms of phosphate control in filamentous fungi and their regulation by external stressing factors are discussed (Section 2), the second part of the article (Section 3) covers the molecular mechanism of calcium uptake and signaling, and finally Section 4 revises the interaction between phosphate and calcium regulation at the cell biology level, including a comparison of the roles of vacuoles and acidocalcisomes in these processes.

BOX 1 **Examples of cross talking between phosphate concentration and calcium homeostasis in filamentous fungi.**

1- A significant part of calcium in vacuoles, endoplasmic reticulum, and other organelles is stored forming complex with polyphosphate (Bootman et al., 2001; Martín and Liras, 2021a).
2- Inhibition of the *Aspergillus fumigatus* calcineurin activity increases expression of six putative phosphate transport genes (Silva-Ferrieiro et al., 2007).
3- Mutants of *Aspergillus fumigatus* defective in the calcineurin catalytic subunit A are impaired in phosphate transport. This effect is reversed by addition of high phosphate concentration (Silva-Ferreira et al., 2007).
4- The phosphate/sodium symporter Pho89 is controlled by the CrzA transcriptional regulator that governs calcium metabolism (Serrano et al., 2002; Serra-Cardona et al., 2014).
5- The transient receptor potential calcium channel CaPhm7 of *Candida albicans*, homologous of the ScPhm7 in *Saccharomyces cerevisiae*, controls expression of the Pho89 symporter (Jiang and Pan, 2018).
6- High intracelullar ATP concentration iresults in the formation of inositol polyphosphate derivatives IP3, IP7 and higher members of this family. IP3 determines the release of calcium from internal stores to the cytosol.
7- Some fungi contain acidocalcisomes organelles that accumulate large amounts of orthophosphate, pyrophosphate and polyphosphate combined with calcium and monovalent cations. Acidocalcisome-like vacuoles are present also in *Saccharomyces cerevisiae* (Seufferheld et al., 2011; Kikuchi et al., 2014; Vila et al., 2022).
8- Phosphorylation of the IP3R enhances calcium release mediated by IP3 (Betzenhauser, and Yule, 2010).
9-The activity of calcium/calmodulin-dependent protein kinases is modulated by protein phosphorylation in response to the intracellular calcium concentration (Tamuli et al., 2011; Jiang et al., 2023).

## 2 Phosphate control of fungal metabolite biosynthesis

Phosphorous in the form of inorganic (Pi) and organic phosphate is an essential nutrient for all living being. Phosphate is a cellular component of nucleic acids, ATP, cyclic AMP (cAMP), and other nucleotides, highly phosphorylated inositol-derivatives and phospholipids. It plays an important role in the oxidative phosphorylation and respiratory chains. Polyphosphate is a phosphate and energy nutrient reserve and polyphosphate synthesizing and solubilizing enzyme systems exerted a relevant function in the growth of mycorrhiza and mycorrhiza-associated plants (Das et al., 2022). *Penicillium oxalicum* has been reported to be very efficient in the release of inorganic phosphate from complex phosphate salts in soil, so called rock phosphate, and this fungus may be used as biofertilizer (Wang et al., 2021). In addition, phosphate participates in numerous regulatory and signaling mechanisms mediated by phosphorylation/dephosphorylation of proteins.

Phosphate regulates the biosynthesis of hundreds of antibiotics in bacteria and filamentous fungi. Early studies in filamentous fungi (Martín, 1977; Martín and Demain, 1980) showed that phosphate inhibits the biosynthesis of important bioactive metabolites. One example is the phosphate inhibition of ergot alkaloids in *Claviceps purpurea* that is exerted at the dimethyl-allyltryptophan synthesis level. The first step of the alkaloid pathway (Rao and Gupta, 1975). Later, it was established that the penicillin biosynthesis in *P. chrysogenum* is also regulated by the phosphate concentration in the culture medium in concert with carbon catabolite regulation (Martín et al., 1999). The concerted action of glucose on phosphate regulation of fungal metabolism is supported by the finding that the phosphate sensing and uptake in *S. cerevisiae* requires the presence of glucose in the culture medium even though this sugar is sensed and transported by systems different from the phosphate transporters (Giots et al., 2003); the interaction of glucose sensing and transport with phosphate uptake is an interesting example of coordination between nutrients assimilation in benefit of fungal metabolism, although the precise mechanism of this interaction needs to be further studied. However, in spite of the relevance of phosphate in bioactive secondary metabolites production there was very little progress until the end of the 20th century. Many of these metabolites are secreted and are known to be associated with the formation of sclerotia and sexual and asexual spores (Lim and Keller, 2014; Mulinti et al., 2014; Calvo and Cary, 2015) but there is little information about whether the biosynthesis of most of these metabolites is regulated by phosphate at the transcriptional, translational or posttranslational level. Parallel studies in bacteria achieved significant progress on the characterization of mechanisms of control of secondary metabolites biosynthesis by phosphate in *Streptomyces* and related actinobacteria (Sola-Landa et al., 2003; Martín, 2004).

### 2.1 The PHO regulon in fungi

In fungi the PHO regulon includes genes that respond to phosphate starvation or sufficiency; initially it was reported that the PHO regulon in *S. cerevisiae* is formed by 22 genes, nine of which are regulatory genes (Ogawa et al., 2000). Similarly, nine genes homologous to those of the yeasts PHO regulon were identified later in the genome of *Aspergillus fumigatus* (Serra-Cardona et al., 2014), however, recent studies have reported a larger number of genes controlled by phosphate in the genome of some fungi (Tomar and Sinha, 2014). A study of *Fusarium graminearum* phosphatome identified up to 82 phosphatase genes (Yun et al., 2015). Eleven of these genes were found to be essential but the remaining 71 phosphatase genes have also impact in fungal development. These phosphatases were shown to be involved in fungal growth and differentiation, in the biosynthesis of secondary metabolites, pathogenesis and virulence.

The proteins encoded by the PHO regulon genes are located in the plasma membrane, the periplasmic space, vacuoles, mitochondria or the cytosol. Several genes were found to encode proteins for scavenging phosphate from the extracellular medium, for polyphosphate hydrolysis and transport across vacuole or mitochondria membrane systems (Persson et al., 2003). Phosphate transport and metabolism are important factors in fungal virulence and pathogenesis. Mutants of *Cryptococcus neoformans* defective in phosphate acquisition and storage showed altered virulence and pathogenicity associated with the production of bioactive metabolites (Kretschmer et al., 2014).

Particularly relevant was the finding in fungi of genes encoding proteins for the transport of inorganic phosphate and regulatory genes that control inorganic phosphate homeostasis. Two of the PHO regulon proteins, encoded by *pho4* and *pho2*, are master regulators that control expression of many *pho* genes. The *S. cerevisiae* Pho4 is a 312 amino acids transcriptional regulator that belongs to the basic helix-loop-helix (bHlH) family (Persson et al., 2003). Pho4 is a dimer that works in collaboration with Pho2 controlling the transcription of the PHO regulon genes and its action is modulated by phosphorylation at serine-threonine residues, that is catalysed by Pho85/Pho80 cyclin-kinase complex (see below) (Komeili, 1999; Secco et al., 2012a; Tomar and Sinha, 2014). Initial studies on the control of gene expression by this transcriptional regulator was made by studying the expression of *pho5*, a gene that encodes the yeast secreted acid phosphatase, an easy to measure reporter of the Pho4-mediated regulon genes. Two activating sequences Uasp1 and Uasp2 that bind Pho4 were found in the region upstream of the *pho5* promoter (Rudolph and Hinnen, 1987; Ogawa et al., 2000). The binding sequences of the Pho4 protein are CACGTG or CACGTT, the Uasp2 sequence is protected by the nucleosome structure and therefore only the Uasp1 sequence is accessible unless the chromatin is remodelled by different protein modifications including acetylation/deacetylation or phosphorylation (Steger et al., 2003). Pho4 binding sequences have been found in almost all the promoters of the PHO regulon genes (Ogawa et al., 2000). Pho4 plays also an important role in the control of pathogenicity and virulence of pathogenic fungi. Studies using a *C. neoformans* mutant defective in Pho4 showed that this regulatory protein is required for phosphate uptake, growth and *C. neoformans* dissemination into the brain (Lev et al., 2017; Bhallan et al., 2022).

### 2.2 Inositol pyrophosphates signaling molecules as reporters in phosphate homeostasis

The mechanism by which fungal cells sense inorganic phosphate is poorly characterized. Inositol pyrophosphates are a family of metabolic messengers characterized by the presence of one or more pyrophosphate (PPi) groups attached to a *mio*-inositol ring (Wera et al., 2001). The presence of energy rich phosphoanhydre bonds in these molecules is one of the characteristic features that explain their important role in regulation of phosphate metabolism. The intracellular messenger IP3 (1,4,5 inositol-triphosphate) is formed by phospholipase C that cleaves phosphatidyl-inositol-4,5 diphosphate yielding IP3 and diacylglycerol. In yeasts, higher members of this family e.g. *mio*-inositolheptakisphosphate (IP7) are formed by sequential phosphorylation of IP3 by pyrophosphate kinases and higher members that contain more phosphate groups than carbon atoms reflects the energetic and phosphorylation status of the cell. Particularly, the ATP concentration required for the conversion of *mio*-inositol hexakisphosphate (IP6) to IP7 since the IP6 kinase has a Km for ATP in the millimolar range (Azevedo and Saiardi, 2017).

In this respect an important finding was the observation that the intracellular concentration of inositol pyrophosphate changes in response to the phosphate concentration in the medium. Proteins involved in cellular reactions of phosphate metabolism, e.g. phosphate transporters, phosphate signaling proteins or polyphosphate polymerases contain a so called SPX domain that interact with inositol pyrophosphates signaling molecules (Secco et al., 2012b). These domains consist of 135–380 amino acid residues and are located in the amino terminal region of the corresponding protein. Initial studies showed that the SPX domain serves as substrate for the interaction of inositol pyrophosphate signaling molecules in plants and animals. In *S. cerevisiae* the *pho90* and *pho87* phosphate transporters interact with the SPL2 protein at its SPX domain and this decreases the phosphate uptake (Wykoff et al., 2007; Hürliman et al., 2009). Three SPX domains of different organisms, including *S. cerevisiae* (Hothorn et al., 2009) were crystalized, providing evidence of the SPX surface that interacts with inositol pyrophosphate (Samyn et al., 2012; Wild et al., 2016). Furthermore, IP7 and higher inositol pyrophosphates act as donors of orthophosphate in protein modifications catalysed by pyrophosphorylases that result in the introduction of an additional phosphate group in the serine phosphate residues in proteins to form serine pyrophosphate units. Therefore, increments in the ATP levels of the cell raises the intracellular level of IP7. In summary, inositol pyrophosphate molecules communicate the inorganic phosphate extracellular level to the SPX domain of the phosphate transporters and other proteins involved in phosphate metabolism, regulating their activity.

### 2.3 Mechanisms of phosphate sensing and transport in fungi

Phosphate availability is extremely important to fungi for growth. Inorganic phosphate is taken up in yeasts and filamentous fungi by the high affinity transporters systems encoded by *pho84* and *pho89* (Bun-ya et al., 1991; Martínez and Persson, 1998; Persson et al., 1999) and low affinity transporters encoded by *pho87* and *pho90* (Bun-ya et al., 1996; Ghillebert et al., 2011; Wykoff and O´Shea, 2001). When the phosphate concentration in the medium approaches the Km of the low affinity system then the cells trigger the formation of high affinity transporters. This provides a time lapse for the cell to survive using the phosphate low affinity transport system until the high affinity transport is fully induced. The importance of phosphate availability in fungal nutrition is highlighted by the presence of five phosphate transport genes *pho84, pho87, pho89, pho90* and *pho91* in *S. cerevisiae*; only quintuple mutants defective in the five transporters are unable to grow, indicating that all of them are active phosphate transporters (Wykoff and O’Shea, 2001).

Analysis of the hydrophobicity of the encoded proteins in yeast showed that both Pho84 and Pho89, belong to the multiple facilitator superfamily (MFS transporters) and have 587 and 574 amino acids respectively, have 12 transmembrane spanning domains with an intervening hydrophilic loop of 74 amino acids between the transmembrane domains TMS6 and TMS7 in Pho84, and a loop of 110 amino acids located between TMS7 and TMS8 in Pho89 (Bottger and Petterson, 2002; Persson et al., 2003). The MFS proteins include many transporters for several primary or secondary metabolites that are involved in communication between cells in pure cultures and/or between different species (Martín et al., 2005). Pho84 and Pho89 are symporters that introduce inorganic phosphate and either protons or Na+, respectively. The *S. cerevisiae* Pho89 high affinity symporter is functionally similar to other sodium transporters in mammals and *Neurospora crassa* (Bottger and Petersson, 2002). The similarity between Pho84 and Pho89 sequences is low (15% amino acids identity) and there are important differences between them. Pho84 has an optimal pH of 4.5 while the optimal pH for Pho89 is 9.5 (Persson et al., 2003). These transporters are under strict phosphate control and are only derepressed at low phosphate concentration. In addition to its control by phosphate starvation, expression of *pho84* and *pho89* increases at alkaline pH, particularly the expression of *pho89* is enhanced by alkaline pH even in mutants defective in the transcriptional activator Pho4 (Lamb et al., 2001; Serrano et al., 2002). The evolutive acquisition of high affinity and low affinity transport systems appears to confer metabolic advantage to yeasts and filamentous fungi (Levy et al., 2011) as it is also well documented in bacteria (Martín and Liras, 2021a).

### 2.4 Transport related nutrient effectors: Pho84

Transport related nutrient effectors (named transfectors) have been discovered in *S. cerevisiae* and in several other eukaryotic cells. These proteins serve as nutrient sensors and are also involved in the nutrient transport mechanisms. In *S. cerevisiae* Pho84 is a transfector that is involved in phosphate sensing and transport, and as effector rapidly activates the protein kinase A pathway in concert with glucose (Giots et al., 2003). Arsenate, is a non-metabolizable structural analogue of phosphate that inhibits growth of fungi suggesting that arsenate binds to phosphate receptors. Mutants defective in the high affinity Pho84 transporter are unsensitive to arsenate suggesting that Pho4 is involved in both phosphate sensing and transport into the fungal cells (Giots et al., 2003; Wykoff et al., 2007; Popova et al., 2010). Phosphate containing molecules that interact with the Pho84 transporter can trigger the phosphate signaling without been transported, as is the case with glycerol-3-phosphate (Popova et al., 2010). These compounds behave as phosphate agonists of Pho84 and, therefore, affect the signaling activity of phosphate. In summary, this information indicates that agonists exert their function by interacting with the Pho84 phosphate binding site but do not require a complete transport into the cell.

### 2.5 Characteristics of the Pho89 phosphate transporter: evidence for interaction of phosphate and calcium metabolism

Remarkably, the Pho89 symporter is under the control of a calcium TRP (Jiang and Pan, 2018) providing evidence of a close interaction between calcium transport and phosphate regulation. The Pho89 phosphate transporter in yeasts is a high affinity transporter functionally similar to the Pit transporter in bacteria (Martín and Liras, 2021) and to the mammalian Na^+^ P3, and is conserved from bacteria to vertebrates (Werner and Kinne, 2001). This protein has 10–12 transmembrane domains, depending on the species, and a large hydrophilic loop between regions TMS7 and TMS8 that has been shown to be located in the cytosolic side of the cell membrane (Chien et al., 1997; Persson et al., 1999). The Pho89 protein of *S. cerevisiae* has a Km for Pi of between 0.4 and 0.6 μM. This transporter is active at alkaline pH but lacks affinity for the substrate at pH below 4.5 in contrast to the proton dependent Pho84 that is very active at a wide pH range (Martínez and Persson, 1998).

In *S. cerevisiae* the Pho89 encoding gene is governed by a Crz zinc finger type transcriptional factor that is activated by the calcium-calcineurin system; accordingly, the *pho89* gene is no expressed in *crz1* mutants (Serrano et al., 2002). Notably, in *S. cerevisiae* expression of *pho89* transporter gene is controlled by Crz1 transcriptional activator of calcium metabolism genes under conditions of alkaline pH providing evidence for another interaction between phosphate metabolism and calcium homeostasis (Serra-Cardona et al., 2014, Box 1). These findings agree with recent observations in *C. albicans*, in which the calcium permeable stress gated CaPhm7 affects the expression of *pho89* (Jiang and Pan, 2018). These results suggests that Pho89 has a clearly distinct role than Pho84 and connects phosphate transport with calcium metabolism (Secco et al., 2012a).

### 2.6 Signaling molecular mechanism and circuits of control regulated by the Pho4 transcriptional factor

**FIGURE 1:**
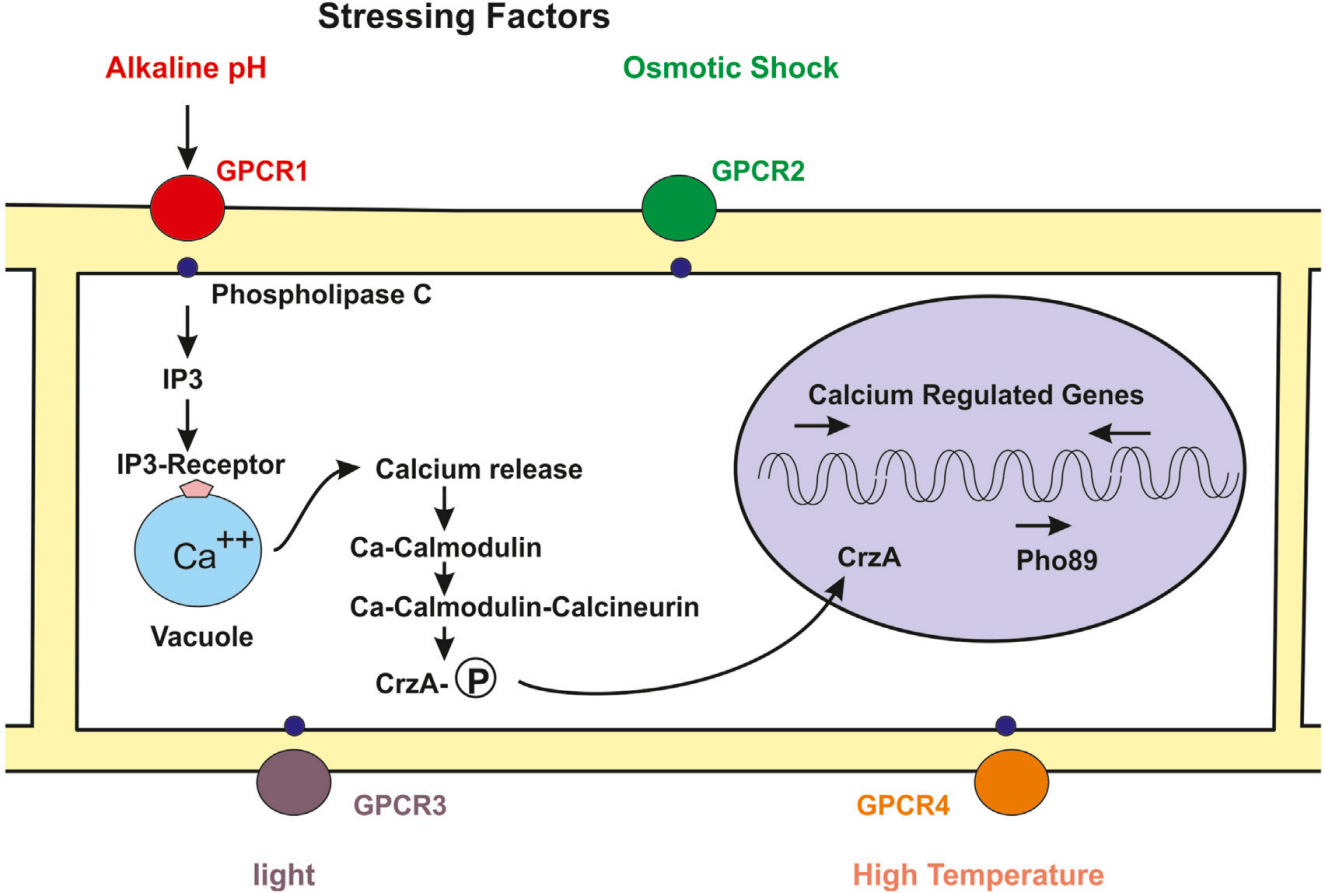
Skeme of the molecular mechanism of regulation of fungal metabolism by the calcium-calmodulin-calcineurin pathway. Numerous stressing signals interact with GPCRs receptors in the cell membrane (circles red, green, purple, orange). The GPCR bind to the phospholypase C (small blue circles) and this protein forms IP3. The binding of IP3 to its receptor in the vacuole (blue clear circle) membrane triggers the release of calcium to the cytosol. Free cytosolic calcium binds to calmodulin and the calcium-calmodulin complex activates the calcineurin phosphatase activity. The phosphatase activity removes phosphate from the phosphorylated Crz1 transcriptional factor and the unphosphorylated Crz1 protein enters into the nucleus (pink circle) and activates expression of the high affinity phosphate *pho89* transport gene and of numerous genes regulated by calcium.

The mechanisms of regulation of phosphate transport and metabolism by the master transcriptional regulator Pho4 are well documented. As indicated above the activation or inactivation of the *pho* genes takes place by phosphorylation or dephosphorylation of the Pho4 regulator. In phosphate sufficient conditions Pho4 is phosphorylated by protein kinases and then excluded from the nucleus to the cytosol, therefore, the PHO regulon genes remain unexpressed (Secco et al., 2012a). In phosphate depleted conditions Pho4 is unphosphorylated, binds to a nuclear import receptor and is introduced in the nucleus where attach to response elements (PRE) and activates different genes of the PHO regulon, including *pho84* and *pho89* (Mouillon and Persson, 2006; Tomar and Sinha, 2014). The phosphorylation of Pho4 is exerted by the cyclin-dependent kinase (CDK) complex Pho80-Pho85. This CDK complex phosphorylates serine/threonine residues in different sites of the Pho4 transcriptional factor and this causes distinct cellular localization (Komeili, 1999; Secco et al., 2012a; Secco et al., 2012b). The activity of the cyclin-dependent kinase complex CDK is controlled by Pho81, which is a CDK inhibitor. Under phosphate limiting condition the cyclin-dependent protein kinase complex is inhibited, resulting in a unphosphorylated active Pho4 and thus the genes of the PHO regulon are activated; *vice versa*, under phosphate sufficiency conditions the Pho81 activity is null and the cyclin-dependent protein kinase is active, resulting in a phosphorylated Pho4 that remains in the cytosol, and therefore the PHO regulon genes are not expressed (Schneider et al., 1994; O´Neill et al., 1996) (Figure 1).

In addition to phosphorylation by the CDK complex the action of Pho4 is affected by trans-acting factor; they include the cooperation with the Pho2 homeodomain (Ogawa et al., 2000; Zhou and O´Shea, 2011). According to these authors Pho2 bind to a sequence distant 15 bp from the Pho4 binding site. When attached to this position Pho2 exert an activation of Pho4 binding to its target DNA. Furthermore, the interaction of Pho4 with its target sequence is controlled by the nucleosome in that DNA region. In *S. cerevisiae* nucleosome depleted regions there is a competition with the Cbf1 factor that recognize the same DNA sequence as Pho4, the relative abundance in the nucleus of these two proteins, Pho4 and ScBf1, determines the occupancy of the Pho4 binding site in the DNA and therefore the expression of the PHO regulon genes (Zhou and O´Shea, 2011). Detailed studies of the genes encoding enzymes of the PHO regulon have been performed only in a few model filamentous fungi, for example *N. crassa* (Tomar and Sinha, 2014). The core of the PHO regulon enzymes is conserved in the studied yeasts and most filamentous fungi but there are significant differences among them. The designation of functionally similar genes is sometimes different, e.g. the core PHO regulon genes in *N. crassa* are named *nuc-1, preg, pgov* and *nuc-2* that correspond to the equivalent *pho4, pho80, pho85* and *pho81* genes of *S. cerevisiae*, respectively (Peleg et al., 1996; Gras et al., 2007; Tomar and Sinha, 2014). Although the core components of the *S. cerevisiae* PHO regulon are conserved in *N. crassa* it is unclear whether additional components occur in other filamentous fungi.

## 3 Calcium in fungi metabolism, an overview

Calcium is an essential nutrient that plays very important roles in fungal metabolism, affecting growth and differentiation (Tisi et al., 2016). Calcium ions serve as intracellular messengers common to all eukaryotic cells. Several extracellular signals, including divalent cations, lithium, ethanol, caffeine, some nitrogen sources, stressing pH and temperatures and antifungal drugs increase the intracellular calcium concentration (Thewes, 2014).

Initial studies on the regulation of calcium metabolism were performed in *S. cerevisiae* and *N. crassa*. At the end of the last century research work on the model filamentous fungi *N. crassa,* showed that fungi establish a calcium gradient in the hyphae that is important for apical fungal growth and development (Jackson and Heath, 1993; Kothe and Free, 1998), and for the secretion of secondary metabolites (Prokisch et al., 1997). In addition, calcium affects the production of bioactive secondary metabolites in many filamentous fungi. One example is the control by calcium of the production of penicillin and the expression of the penicillin biosynthetic genes (Domínguez-Santos et al., 2017). Vacuoles, peroxisomes and related microbodies involved in penicillin biosynthesis and secretion are concentrated in a hyphal region determined by the calcium gradient (reviewed by Martín and Liras, 2022b).

### 3.1 Tolerance to calcium stress: the neuronal calcium sensor

Calcium is taken up by the cells using different P-type membrane calcium translocases and calcium ATPase pumps (reviewed by Espeso, 2016). Excessive accumulation of calcium in the cells creates a calcium stress problem since high intracellular concentration of calcium interfers with nucleotide, polyphosphate and other macromolecules function. To avoid the calcium toxicity fungi and other eukaryotic cells have a system for calcium tolerance/sensitivity, the neuronal-calcium sensor (NCS). Transient increase in calcium causes activation of various Ca^2+^ binding proteins, including the neuronal calcium sensor-1 (NCS-1). Members of the NCS family contain an N-terminal myristoylation domain and four Ca^2+^ binding EF-hand domains (Burgoyne et al., 2004; Burgoyne, 2007). The NCSs are small proteins evolutively conserved across the species, and homologue proteins have been identified in yeast, filamentous fungi and mammals among other living beings (Sánchez-Gracia et al., 2010; Tamuli et al., 2011) In *S. cerevisiae* the NCS-1 protein is essential for growth and it is involved in the regulation of the activity and cellular location of the phosphatidyl-inositol-4-kinase (Huttner et al., 2003; Strahl et al., 2007). A knock-out mutant of *N. crassa* NCS-1 shows increased sensitivity to high calcium concentration and UV light (Tamuli et al., 2011) suggesting that NCS-1 is involved in calcium tolerance in this fungus. Similar increase sensitivity to calcium concentration has been observed in a NCS mutant of *Magnaporthe grisea* (Saitoh et al., 2003); however, the NCS null mutant of *A. fumigatus* does not show increased sensitivity to calcium concentration, indicating that the behaviour of NCS in *A. fumigatus* is different from that of other fungi (Mota et al., 2008). The NCS-1 homologous of *A. fumigatus* (NCS-A) is not essential for growth but plays an important role in sterols distribution in membrane domains and polar growth (Mota et al., 2008). The mechanism of NCS-mediated stress tolerance has been studied in *N. crassa* (Gohain and Tamuli, 2019). Increasing calcium levels enhances expression of the NCS-1 encoding gene. Importantly, these authors observed that the expression of the NCS-1 encoding gene is controlled by the calcineurin responsive zinc finger Crz1 transcriptional regulator (see below) indicating that there is a clear regulation of the NCS-1 protein by the calmodulin-calcineurin signaling pathway.

### 3.2 The calcium sensor calmodulin and its interaction with proteins

Intracellular calcium binds to calmodulin, a protein encoded by the *cmd1* gene (Cyert, 2001). Calmodulin is a small protein, 17 kDa, that is highly conserved in all eukaryotic organisms, and binds Ca^2+^ ions with high affinity (Kd 10^−6^ to 10^−5^ M). In *S. cerevisiae* it contains four amino acid motives, named EF-hands for calcium binding, although only three of the EF-hands in the calmodulin molecule are functional, the fourth lacks the Ca^2+^ binding loop (Kraus and Heitman, 2003). These motives consist in 12 amino acid loops that connect alpha helices and contain glutamic acid and aspartic acid residues that chelate the Ca^2+^ ion (Davis et al., 1986; Cyert, 2001).

Calmodulin, usually activated by Ca^2+^ acts by at least three different mechanisms: 1) interacting directly with proteins involved in the biosynthesis of bioactive metabolites, differentiation and pathogenicity, 2) Activating the calcium-calmodulin complex a class of protein kinases named calcium/calmodulin-dependent protein kinases, and 3) binding to the protein calcineurin what triggers the calmodulin-calcineurin cascade that regulates numerous genes in the cell (Figure 1).

### 3.3 Direct interaction of calmodulin with secondary metabolites biosynthetic enzymes

Calmodulin interacts directly with many proteins in fungal cells. In the calcium-dependent processes calmodulin binds calcium through the EF hands what results in a change of calmodulin configuration that modulates its interaction with other proteins.

**FIGURE 2:**
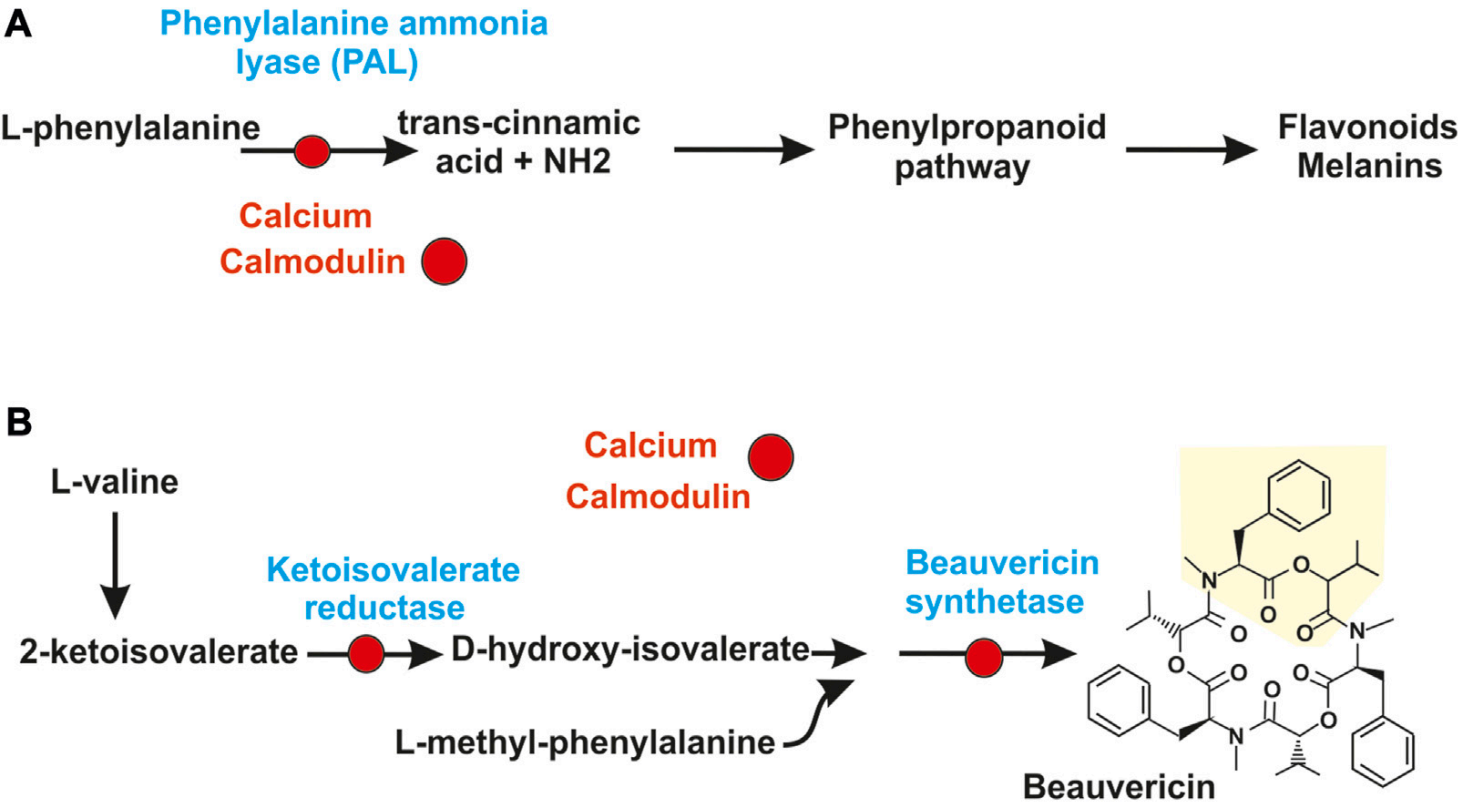
Calcium control of secondary metabolites by direct interaction of calmodulin with secondary metabolites synthesizing enzymes. **(A)** Regulation by calcium of the phenylpropanoid pathway. The calcium-calmodulin complex (red circles) binds and inactivate the phenylalanine ammonia lyase (PAL), first enzyme of the phenylpropanoid pathway. This inactivation blocks the pathway and the formation of secondary metabolites derived from it, as flavonoids and melanins. **(B)** Skeme of biosynthesis of beauvericin in *Beauveria bassiana*. The calcium-calmodulin complex (red circles) binds and inactivate: 1) the 2-ketoisovalerate reductase converting 2-ketoisovaleric acid in d-hydroxyisovalerate and 2) the non-ribosomal peptide synthetase of *Beauveria bassiana* that condenses three units of L-methyl-phenylalanine-d-hydroxyisovalerate depsipeptide to form beauvericin.

The direct interaction of calmodulin with secondary metabolites synthesizing enzymes has been studied in the insect pathogen *Beauveria bassiana*; this fungus produces several secondary metabolites, including compounds derived from the phenylpropanoid pathway and non-ribosomal peptides among others. The phenylpropanoid pathway starts from phenylalanine that is converted to cinnamic acid by the phenylalanine ammonia lyase (PAL) or a related tyrosine ammonia lyase (TAL) that forms coumaric acid (Figure 2A) (Martín and Liras, 2022a). Calmodulin interacts directly with the PAL enzyme of *B. bassiana* and inhibits its activity (Kim et al., 2015). Other interesting example is the formation of beauvericin, a product of *B. bassiana* with antimicrobial, antiviral and insecticidal activities. One structural component of beauvericin is 2-hydroxyisovaleric acid that is formed from 2-ketoisovaleric acid by the 2-ketoisovaleric acid reductase. Then a 325 kDa non-ribosomal peptide synthetase (NRPS) forms beauvericin (Figure 2B). Calmodulin binds the 2-ketoisovalerate reductase *in vivo* and *in vitro* and inhibits the biosynthesis of this precursor and of the final product, beauvericin (Kim et al., 2016). Salt stress and light inhibit the biosynthesis of 2-ketoisovalerate and beauvericin and this inhibition is reversed by calmodulin inhibitors indicating that the effect of these stressing factors is mediated by the interaction of calmodulin with the 2-ketoisovalerate reductase. In addition, calmodulin interact directly with the beauvericin synthetase. Calmodulin binding sequences have been found in the PAL, the 2-ketoisovalerate reductase and the beauvericin synthetase of *B. bassiana* (Figure 2) and similar sequences occur in many calmodulin binding proteins (Kim and Sung, 2018). The calmodulin binding domain consist in stretches of 16–35 amino acids that form a basic amphipathic α-helix (O´Day, 2003). The beauvericin synthetase contains a calmodulin-binding domain in the C-terminal region; binding of calmodulin to this region was confirmed by experiments using a synthetic binding domain and the formation of a complex was demonstrated by non-denaturing polyacrylamide gel electrophoresis (Kim and Sung, 2018). Formation of this complex takes place in presence of calcium ions but not in their absence. The direct interaction of calmodulin with enzymes involved in the biosynthesis of bioactive metabolites is of great interest although much more research needs to be done to advance in this field.

### 3.4 Calcium and calmodulin-dependent protein kinases

Very small increases in cytosolic calcium content triggers the rapid formation of several protein kinases, including the calcium/calmodulin-dependent protein kinases (named CamKs). These enzymes are serine/threonine protein kinases that are involved in distinct mechanisms of control of calcium homeostasis in eukaryotic cells. Most of these calcium/calmodulin dependent protein kinases have been characterized in mammals but there is little information of this group of proteins in filamentous fungi (Tamuli et al., 2011). Studies on the structure of these proteins indicate that they contain an amino terminal protein kinase domain linked to an autoinhibitory domain that overlaps with the calmodulin-binding domain (Hook and Means, 2001). These enzymes show an elaborated mode of action: the autoinhibitory domain binds to the catalytic centre and maintains the protein kinase in an inactive state until calcium and calmodulin bind to the calmodulin binding site and, therefore, activate the protein kinase activity. These calcium/calmodulin protein kinases differ in their range of substrate specificity (Tamuli et al., 2011). Protein kinases of this class were found in *S. cerevisiae*, *Schizosaccharomyces pombe* (Melcher and Thorner, 1996; Rasmussen, 2000) and also in several filamentous fungi (Table 1). In *N. crassa* two different CamKs protein kinases have been identified that regulate the circadian clock (Yang et al., 2001) and sexual development of ascospores (Tamuli et al., 2011). Recently, three calmodulin-dependent protein kinases have been studied in *Alternaria alternata*, a fungus that causes pears infection. Gene disruption studies indicated that mutation of one of the kinases gene does not affect the overall growth but reduces hyphal differentiation, sporulation and melanin formation (Jiang et al., 2023). Expression of the three kinases is intense during the infection process; in addition inhibition of CamKs activity supress the infection process indicating that these kinases play key roles in pathogenicity. Some of these enzymes are also important in the development of invasion structures in pathogenic fungi. In *C. gloeosporioides* an inhibition of the CamKs delays spore germination and formation of the appressorium. Treatment of *C. gloeosporioides* with inhibitors of these protein kinases reduces also the formation of melanin associated with the penetration of the appressorium during infection of the hosts (Kim et al., 1998). The available information suggests that there are different roles played by these calcium/calmodulin protein kinases in fungi. In summary, phosphorylation of proteins by these calcium calmodulin-dependent protein kinases provides a novel example of interaction between calcium homeostasis and phosphate regulation (Box 1).

**TABLE 1:** Documented examples of calcium/calmodulin-dependent protein kinases in fungi.

Fungi/yeast	Strain relevance in infection/Pathogenicity and biosynthesis of bioactive metabolites	References
*Saccharomyces cerevisiae*	Model budding yeast	Melcher and Thorner (1996)
*Colletotrichum gloeosporioides*	Plant infection, appressorium development, spore germination	Kim et al. (1998)
*Magnaporthe grisea*	Rice blast fungus. Appressorium development, spore germination	Lee and Lee (1999)
*Schizosaccharomyces pombe*	Model of fision yeast	Rasmussen (2000)
*Aspergillus nidulans*	Model filamentous fungus. Production of mycotoxins (sterigmatocystin) and penicillin G	Joseph and Means (2000)
*Neurospora crassa*	Model filamentous fungus. Ascospores formation	Zelter et al. (2004); Kumar and Tamuli (2014)
*Sporothrix schenckii*	Producer of sporotrichosis in humans. Melanin production	Valle-Aviles et al. (2007)
*Colletotrichum falcatum*	Plant infection, appressorium development, spore germination	Karunakaran et al. (2006)
*Phyllosticta musarum*	Freckle disease of banana	Wang et al. (2008)
*Magnaporthe oryzae*	Rice plant infection, appressorium development, spore germination	Liu et al. (2009)
*Setosphaeria turcica*	Causal agent of northern corn leaf blight in maize	Wu et al. (2015)
*Arthrobotrys oligosporus*	Nematode-trapping fungus	Zhen et al. (2019)
*Pseudopeziza medicaginis*	Fungal pathogen of alfalfa lives	Ma et al. (2020)
*Alternaria alternata*	Plant pears infection. Melanine production	Jiang et al. (2023)
*Puccinia striiformis*	It causes stripe rust on wheat	Jiao et al. (2017)

Calcium/calmodulin dependent protein kinases occurs in sequenced genomes of many other fungi.

### 3.5 The calmodulin-calcineurin signaling cascade

Fungi sense different nutrients in the medium and transduce the signal by cascades that produce adequate responses in the cell, some of them mediated by the calmodulin-calcineurin pathway (Martín et al., 2019). In contrast with the little information available on regulation of fungal metabolites by direct interaction of calmodulin with the antibiotic synthetases, the molecular mechanism of calcineurin-mediated regulation has received much attention in several fungi. Calcineurin is a heterodimeric protein phosphatase that consist in a catalytic subunit CnA and a regulatory subunit CnB. The catalytic subunit CnA contains the phosphatase active center and an associate regulatory region which are separated by a small amino acid stretch. This regulatory region serves to link together the CnA and CnB subunits and, in addition, contains domains for interaction with calmodulin and for self-inhibition (Klee et al., 1998). Calcineurin is the target of the immunosuppressants cyclosporin and FK506 (tachrolimus) and these compounds have been extensively used in research to block the activity of calcineurin *in vivo*. The calcineurin in *S. cerevisiae* regulates expression of numerous genes through the transcriptional factor Crz1 (calcineurin responsive zinc finger). For example, it regulates cell wall biosynthesis, ion homeostasis, vesicles traffic and protein degradation (Yoshimoto et al., 2002).

The calmodulin-calcineurin signaling pathway in filamentous fungi and yeasts is triggered by external stressing factors that are recognized at the membrane level by G protein couple receptors (GPCR) (Krishnan et al., 2012; Martín et al., 2019). Some phospholipases, particularly phospholipase C and the secretory phospholipase La2 (SPLa2) exert important roles in calcium homeostasis (Barman and Tamuli, 2015). In mammals, the GPCRs activate the membrane phospholipase C that forms inositol-1,4,5 triphosphate (IP3, so-called calcium releasing factor); this factor interacts with specific receptors resulting in cytosolic accumulation of Ca^2+^. Interaction of IP3 with the IP3 receptor (IP3R) changes the configuration of this receptor opening calcium channels (Prole and Taylor, 2011, 2019; Taylor and Machaca, 2019); as a result, calcium is released from the endoplasmic reticulum and is redistributed to the cytosol and different organelles. The IP3 receptors of mammals are phosphorylated by the cAMP-dependent protein kinase A (Taylor, 2017). This phosphorylation of IP3R is another example of the interaction of phosphate modification of proteins in calcium homeostasis. Phosphorylation of the IP3 receptors in humans enhance the IP3-mediated calcium release (Betzenhauser et al., 2010; Taylor, 2017) (Box 1). The IP3 receptor contain an ATP-binding site and it has been shown that ATP regulates IP3-mediated calcium release. In addition to ATP other adenine nucleotides and also GTP appears to exert a regulatory function on IP3R affinity, although the selectivity of the nucleotide binding is still unclear.

#### 3.5.1 IP3 receptors in eukaryotes: do IP3 receptors exist in fungi?

IP3 receptors are expressed in most animal cells and protozoa (Prole and Taylor, 2011). This type of receptors has been described in several insects following the initial identification in *Drosophila melanogaster* (Yoshikawa et al., 1992). The IP3R of insects has about 60% identity to those of mammals suggesting that they are relatively well conserved (Toprak et al., 2021). Enzymes involved in the biosynthesis and turnover of IP3 have been found in endoparasites of the Apicomplexa group; however, there is no clear evidence of the presence of canonical IP3R in these parasites and has been suggested that they may harbour a primitive non-canonical type of IP3 receptor (Garcia et al., 2017). A similar non-canonical IP3R may occur in filamentous fungi, since the biosynthesis of IP3 and other enzymes related to IP3 metabolism occurs in fungi (see Section 2.2); however, additional research is needed to clarify this hypothesis.

The increase of intracellular calcium is sensed by calmodulin which upon calcium binding interacts with the calcineurin activating its phosphatase activity, this start the calmodulin-calcineurin cascade and results in the dephosphorylation of CrzA (Crz1) that is then transported to the nucleus by a nuclear membrane import receptor (Figure 1).

The fungal calcineurin is highly similar to the homologous phosphatase in animal cells, which plays a very important role in their metabolism. Pioneer studies in human cells indicated that calcineurin exert its function by dephosphorylating the nuclear factor of activating T cells (NFAT) what results in introduction of the dephosphorylated NFAT to the nucleus and subsequent activation of the calcineurin-dependent genes (Crabtree, 2001). A similar mechanism was found in filamentous fungi involving the CrzA transcriptional factor.

### 3.6 The calcineurin role in the calcium signaling pathway

In fungi calcineurin works through regulation of different transcriptional factors, of which the best known is Crz1 in *S. cerevisiae,* named CrzA in some filamentous fungi (Stathopoulos and Cyert, 1997). Crz1 contains a C2H2 zinc finger motif that binds to calcineurin-dependent regulatory elements (CDRE) (Fedotova et al., 2017; Gohain and Tamuli, 2019). The nuclear concentration of CrzA changes in a pulsatile mode rather than maintaining a constant level. This means that the CrzA-mediated calcineurin signaling is modulated by the frequency, duration and amplitude of the pulses (Dalal et al., 2014).

Following the identification of Crz1 in *S. cerevisiae*, the *crzA* gene was cloned from *A. fumigatus* and several other filamentous fungi (Soriani et al., 2008). Disruption and complementation of *crzA* in *A. fumigatus* proved that this gene is involved in cell tolerance to high calcium and manganese concentrations. In addition, the *crzA* mutant showed an altered expression of several genes encoding calcium transport at high calcium concentration, and decreased virulence. A detailed characterization of the role of CrzA has been made in *A. nidulans* using *crzA* defective mutants (Hagiwara et al., 2008; Spielvogel et al., 2008) demonstrating that CrzA was the regulator of calcium homeostasis. The *crzA-*defective mutant was highly sensitive to external calcium or manganese concentrations and to alkaline pH. This mutant was also altered in a vacuole calcium exchanger (Hagiwara et al., 2008). Spielvogel et al. (2008) observed that the *crzA* mutant had an aberrant morphology related to decreased expression of the chitin synthetase gene. Interestingly, GFP fluorescent protein labelled CrzA was located in the cytosol when the cells were cultured in low calcium concentration but upon calcium addition was rapidly internalized into the nucleus and therefore exerts its regulation by binding to CDRE sequences (Figure 1). The CrzA gene of *Aspergillus parasiticus* is required for the production of aflatoxin under calcium-stress conditions (Chang, 2008) and in *Fusarium oxysporum* CrzA is needed for the production of deoxynivalenol (DON) and for virulence (Chen L. et al., 2019) (see Section 3.7).

### 3.7 Ambient pH, osmolarity and heat shock stresses effect on the calcineurin mediated regulatory cascade

Several authors have studied the effect of alkaline pH or high calcium concentration on the control of the calmodulin-calcineurin cascade. It is well known that in *S. cerevisiae* alkaline pH triggers a rapid uptake of calcium and the calcium increment in the cytosol triggers the calcineurin mediated cascade (Serrano et al., 2002; Yoshimoto et al., 2002; Viladevall et al., 2004).

The role of *Aspergillus oryzae* calcineurin in response to stress has been studied in detail by Juvvadi et al. (2003). In *A. oryzae* sequences for binding stress response regulatory elements (STRE) and for heat stress factor binding have been found in the upstream region of the *cnA* gene of *A. oryzae*, encoding the calcineurin catalytic subunit CnA**.** The calcineurin activity in the wild type *A. oryzae* increased in response to alkaline pH, salt concentration, and high temperatures. Blocking the *cnA* mRNA by expression of the antisense RNA decreases the activity of the calcineurin under stressing conditions and caused reduced growth. In contrast overexpression of *cnA* resulted in an increased calcineurin activity under stressing conditions and produces tolerance to the calcineurin inhibitor FK506. These results support the conclusion that adaptation to different stressing factors in this fungus is mediated by the calcineurin signaling pathway.

### 3.8 Impact of the calmodulin-calcineurin pathway in expression of genes related to antifungal activity

There are many examples of the effect of stressing factors and alkaline pH on the production of antimicrobial fungal products. One of them is the formation of the cysteine rich antifungal protein AFP that is produced by several species of *Aspergillus* and *Penicillium.* The AFP protein is of interest because it shows antifungal activity against pathogenic yeasts and filamentous fungi (Huber et al., 2018).

The AFP protein is a small protein (6.2 kDa in *P. chrysogenum*) with potent antifungal activity toward *Fusarium* and *Aspergillus* species (Theis et al., 2005), some of which are plant pathogens. Therefore, it has been suggested to use AFP in the protection of vegetables such as tomato, grape and rice plant infections by *Fusarium oxysporum* and *Botrytis cinerea* **.** Expression of the AFP encoding gene is upregulated at alkaline pH (Meyer and Stahl, 2002a; Mayre et al., 2002b) and is abolished by the calcineurin inhibitor FK506 suggesting that transcription of the encoding gene is regulated by the calmodulin/calcineurin cascade (Meyer et al., 2005). This hypothesis is supported by the finding in the upstream region of the AFP encoding gene of a CDRE sequence that is the binding site for the CrzA transcriptional regulator. The mode of action of the AFP protein in relation to calcium metabolism has been elucidated in *N. crassa*. The AFP protein was shown to increase significantly the intracellular calcium concentration of *N. crassa* resting cells. The response of calcium metabolism to the addition of AFP change by chelating the extracellular calcium with EDTA, or other calcium specific chelators; calcium chelation counteract the AFP perturbation of calcium homeostasis indicating that calcium ions are involved and required for the AFP toxicity effect. The calcium transport blocker diltiazem exerts the same effect on calcium homeostasis as the addition of AFP (Binder et al., 2010). The addition of both AFP and diltiazen exert an cumulative effect on calcium homeostasis. In summary, in *N. crassa* the effect of AFP is due to the transport of calcium into the cells or its release from internal stores into the cytosol, where it triggers the regulation of calcium homeostasis, although the molecular mechanism by which AFP affects the calcium sensing and/or transport is not yet clear.

### 3.9 Calcineurin in fungal pathogenesis related to production of bioactive secondary metabolites

The infection and virulence of several animal and plant pathogenic fungi is regulated by calcium. Early studies on calcineurin mediated regulation in the pathogenic filamentous fungi *M. grisea* and *B. cinerea* indicated that calcineurin controls the formation of invasion structures. The development of these structures and the infection in plants is inhibited by cyclosporin (Viaud et al., 2002, 2003; Schumacher et al., 2008). In these infections, calcium regulates growth rate, morphology, differentiation of the filamentous fungus and attachment and penetration in the plant (Gupta et al., 2022). This is a very complex phenomenon that requires many enzymes, and bioactive metabolites that play important roles in intercellular communications (Table 2), however, in only a few fungal infections there is clear evidence of the involvement of calcineurin regulation in secondary metabolites production. Here we describe a few of the more representative examples. *A. parasiticus* produces several toxins, including aflatoxin. Mutants of two strains deficient in *crzA,* grown in media supplemented with calcium produce very low amounts of aflatoxin and O-methylsterigmatocystin. Transcriptional studies of the aflatoxin biosynthetic genes revealed that three of them, *nor1, ver1* and *omtA* were very poorly transcribed in a *crzA* defective mutant indicating that expression of the aflatoxin genes was under the control of the calcium/calcineurin signaling pathway in calcium stressing conditions (Chang, 2008).

**TABLE 2:** CrzA regulation of bioactive secondary metabolites in fungi.

Fungi	Bioactive metabolite	Biological activity	References
*Aspergillus parasiticus*	Aflatoxin	Toxin	Chang (2008)
*Fusarium graminearun*	Trichothecene	Toxin	Wang et al. (2021)
*Fusarium oxysporum*	Trichothecene	Toxin	Chen et al. (2019b)
*Verticillium dahliae*	Melanin	Protective pigment	Xiong et al. (2015)
*Penicillium digitatum*	Melanin	Protective pigment	Zhang et al. (2009)
*Aspergillus fumigatus*	Melanin	Protective pigment	Zhang et al. (2009)
*Cryptococcus neoformans*	Melanin	Protective pigment	Rodrigues et al. (2008)
	Glucuronosyl-manan	Pathogenicity determinant	Rodrigues et al. (2008)
*Alternaria alternata*	AK Toxin	Plant Toxin	Tsai and Chung (2014)
*Neurospora crassa*	Neurosporene, axthaxantine, carotenoid precursors	Protective pigments	Avalos et al. (2013)
			Barman and Tamuli (2015)

An important plant pathogen is *Fusarium graminearum* that produces the *Fusarium* head blight disease in wheat and other cereals (Chen et al., 2019). This fungus synthesizes several trichothecenes which are highly toxic and carcinogenic to humans and other mammals. The trichothecenes include the toxins deoxynivalenol (DON), nivalenol, and zearalenone (Goswami and Kistler, 2004; Pestka and Smolinski, 2005). DON and its acetylated derivatives three acetyl deoxynivalenol and 15 acetyl deoxynivalenol are highly toxic compounds.

Recently, Chen et al. (2019a, b) studied the role of Crz1 of *F. graminearum* and observed that in addition to the effect on growth and differentiation, the Crz1 mediated regulation reduces drastically the trichothecenes formation. Importantly, expression studies of the *F. graminearum crz1* mutant compared to the parental strain revealed that twelve trichothecene *try* genes involved in the biosynthesis of DON have significant reduced expression (Chen et al., 2019). These results connect previous studies on regulation of DON biosynthesis (Hu et al., 2014; Yu et al., 2014; Zheng et al., 2016) with the calcium-calcineurin mediated control of this toxin (Imazaki et al., 2010).

Melanin is a dark pigment that has important roles in protecting the fungal cells against UV irradiation and in the resistance of different microorganisms to antimicrobial agents (Nosanchuk and Casadevall., 2006). In *Verticillium dahliae,* that causes the *Verticillium* wilt in trees, the *crz*A gene is involved in the biosynthesis of microconidia melanin which is important for the pathogenesis of this fungus (Xiong et al., 2015). Similar observations were also made in the mutants defective in *crzA* of *Penicillium digitatum* that also forms melanized microconidia (Zhang et al., 2009). Another important fungal pathogen is the citrus pathogen *Alternaria alternata* that synthesizes the polyketide AK toxin. This fungus contains a PLC-phospholipase similar to the well characterized enzyme of *N. crassa* that forms IP3. IP3 diffuses and binds to IP3 receptors and induces release of calcium ions from the vacuoles. The phospholipase of *A. alternata* is involved in calcium homeostasis, pathogenicity and virulence (Tsai and Chung, 2014).

Another example of calcium-calcineurin Crz-mediated regulation of pathogenicity is the production of virulence factors by *C. neoformans*. This fungus is a basidiomycete that causes meningoencefalitis in humans, a severe disease with high mortality (Fu et al., 2018). During the infection *C. neoformans* release pigments, enzymes and several virulence factors that are secreted in vesicles, one of the major virulence factors is glucuronosyl-manan that is released forming the extracellular capsule, a pathogenicity determinant that has potent immunosuppressive activity (Rodrígues et al., 2008).

In *N. crassa* there are detailed studies on the effect of calcineurin on the sexual and asexual spore development (Tamuli et al., 2016) and on the formation of some secondary metabolites, e.g. carotenoids **(** Barman and Tamuli, 2015 **).** The *N. crassa* carotenoids include the neurosporene astaxanthin and some carotenoid precursors (Avalos et al., 2013) that are responsible of the orange colour of *N crassa.* These carotenoids prevent damage produced by UV light irradiation (Barman and Tamuli, 2015).

### 3.10 Regulators of calcineurin biosynthesis: effect of Rcn regulators on the biosynthesis of secondary metabolites

Calcineurin affects many reactions in the cell and therefore its activity is likely to be regulated by different cell factors. A family of these factors, named calcipressins, bind the calcineurin CnA subunit inhibiting its phosphatase activity (Juvvadi et al., 2014, 2015). In yeasts one of the Crz regulated genes encodes a calcineurin modulator, Rcn1 (RcnA in some fungi), named for regulator of calcineurin, that belongs to the calcipressins family. These are cytosolic proteins that do not enter in the nucleus and, therefore, appear to modulate the calcineurin activity by direct or indirect interaction with this phosphatase. The CrzA protein of *A. fumigatus* affects also expression of some genes involved in different aspects of calcium metabolism (Soriani et al., 2010). *A. fumigatus rcnA* null mutants are affected in the expression of several genes, including the gene for the calcineurin subunit A (Pinchai et al., 2009) and, therefore, these authors proposed that RcnA might exert a feedback regulation of the calcineurin-mediated regulatory cascade.

Members of the Rcn family are conserved in all eukaryotes from yeast to humans (Kingsbury and Cunningham, 2000). Several studies provide evidence on the role of calcipressing in fungal biology includin*g S. cerevisiae* (Mehta et al., 2009) and in the fungi *A. fumigatus, A. nidulans, C. neoformans* and *Magnaporte oryzae* (Pinchai et al., 2009; Liu et al., 2022). The exact mechanism of regulation of calcineurin by Rcn1 is complex. This protein contains a proline-serine repeated motif which seems to be phosphorylated in two different serine residues by the mitogen-activated protein kinase (MAPK). As a consequence of the phosphorylation of Rcn1 the interaction with the calcineurin subunit A is modified and calcineurin phosphatase activity is modulated; when Rcn1 is unphosphorylated the regulator exert a negative effect on calcineurin while when Rcn1 is phosphorylated the regulator does not inhibit the calcineurin phosphatase activity (Hilioti et al., 2004; Kishi et al., 2007; Li et al., 2011).

Recently the mechanism of regulators of calcineurin on the biosynthesis of secondary metabolites has been elucidated in *M. oryzae* *,* a fungus that causes the rice blight disease (Zhang et al., 2016b, 2019). During infection *M. oryzae* attaches to the rice plant cells and form a melanin rich appressorium that penetrates the plant tissue (Zhang et al., 2019). Deletion of the *MoRCN1* gene causes a decrease in virulence and reduces the formation of several secondary metabolites. Transcriptomic analysis of this *M. oryzae* mutant has revealed that the Rcn1 regulator enhances expression of 491 genes and causes downregulation of 377 genes. The Rcn1 regulated genes encode enzymes involved in the biosynthesis of lysine, serine, threonine and the aromatic amino acids, and several genes involved in lipids biosynthesis and fatty acid degradation that may provide precursors for secondary metabolites, including genes for the biosynthesis of staurosporin, indolditerpenoids, meroterpenoids and aflatoxins. The affected genes encode also an ABC transporter; these transporters are frequent in many secondary metabolite gene clusters (García-Estrada et al., 2011; Abu Ammar et al., 2013; Martín, 2020).

## 4 Interaction of phosphate and calcium regulation: vacuoles and acidocalcisomes

Acidocalcisomes are membrane bound acidic calcium store organelles rich in orthophosphate, pyrophosphate and polyphosphate bound to calcium, magnesium and sodium. In these organelles calcium is bound to a polyanionic matrix of polyphosphate although calcium may be released by alkalinization of these vesicles (Docampo and Moreno, 2011). Acidocalcisomes have been found in all kingdoms of life from bacteria to humans. In some bacteria aggregates of polyphosphate (called volutine granules) have been found to be surrounded by a membrane. These vesicles were initially characterized in Trypanosomes but since then they have been studied in different eukaryotic cells including some fungi (Docampo et al., 2010; Seufferheld et al., 2011). Acidocalcisomes have been found in arbuscular mycorrhizae, and acidocalcisome-like vacuoles occur in *S. cerevisiae* (Kikuchi et al., 2014; Vila et al., 2022). Acidocalcisome-like vacuoles are characterized by their biochemical features, mainly acidic character, storage of polyphosphate and calcium ions. One or two proton pumps and transporters maintain the acidity of these organelles including the proton vacuole ATPase in yeasts and/or the vacuole proton pyrophosphatase (VP1) in other eukaryotic cells. The synthesis of polyphosphate and its transport to these organelles is performed by a vacuole transporter chaperone complex (VTC) that is well characterized in yeasts and Trypanosomes (Lander et al., 2016). Regarding calcium transport, acidocalcisomes contain a calcium ATPse involved in calcium uptake and some eukaryotic organisms contain additional ATPses that transport magnesium, zinc, inorganic phosphate and polyamines (Huang et al., 2014). Indeed, four polyamine transporters named TPO1 to TPO4 have been found in the vacuole membrane of *S. cerevisiae* (Tomitori et al., 2001). They belong to the MFS superfamily and contain 12 transmembrane domains. Disruption of the *tpo* genes revealed that these polyamine transporters are involved in uptake of several polyamines and its accumulation in the vacuoles. The disrupted mutants showed increase sensitivity to polyamines, indicating that these transporters participate in polyamine tolerant mechanisms. Noteworthy, fungal vacuoles have similar behaviour as acidocalcisomes, including the ability to acidify vacuoles by calcium ATPses.

Acidocalcisomes are rich in pumps and membrane transporters involved in cation and phosphate homeostasis and calcium signaling. Acidic pH conditions are required to bind these metals to polyphosphate. The biosynthesis of polyphosphate by VTC in filamentous fungi vacuoles and protozoa acidocalcisomes support the proposal that these two organelles are somehow functionally similar. Specialized acidocalcisomes have been found in the tropical pathogenic fungus *Fonsecaea pedrosoi* (Franzen et al., 2008). This fungus contains melanosomes which are membrane bound organelles that contain high levels of polyphosphate, calcium, metals and particularly melanin. The internal pH of eukaryotic melanosomes is very acid and this acidity favours the binding of metals to polyphosphate. The pathogenicity of this fungus is associated to its high level of melanin (see above Section 3.7). Electron microscopy studies, biochemical and immunochemical analysis of the content of melanosomes revealed that melanin is accumulated in sequential stages of development of this structure, suggesting that these organelles are specialized in melanin accumulation (Huang et al., 2014).

## 5 Summary and future outlook

During the last decades it has been established that the biosynthesis of secondary metabolites in filamentous fungi is controlled by elaborated interactions between the phosphate concentration in the culture medium and calcium ions that serve as intracellular messengers in numerous reactions in the fungal metabolism. The biosynthesis of hundreds of secondary metabolites is controlled by phosphate in filamentous fungi and in bacteria; notable advances have been made in our understanding of the mechanisms of phosphate control of secondary metabolites in actinobacteria (Martín and Liras, 2021a) but there is less information on equivalent control in filamentous fungi. Twenty-two genes were initially reported in the PHO regulon in *S. cerevisiae,* nine of them regulatory genes; but many more genes are regulated by Pho4 in some filamentous fungi. Noteworthy, eighty-two phosphatase genes have been reported recently in the genome of *F. graminearum* (Yun et al., 2015)*.* The homeostasis of phosphate in fungi and its role is more elaborate that it was previously reported. The importance of phosphate availability in fungal metabolism is reflected by the presence of two well characterized high affinity phosphate transporters in *S. cerevisiae,* Pho84 and Pho89, and two low affinity transporters, Pho87 and Pho90. Pho84 is a well know transfector (nutrient-related transport effector) that performs both phosphate sensing and phosphate transport (Popova et al., 2010). Some phosphate analogues interact with phosphate receptors exerting a regulatory function although they are not transported into the cells. These findings open new fields for studying the interaction of transporting and sensing mechanisms that should be elucidated in the near future. In contrasts to Pho84, that is a phosphate and proton symporter, Pho89 is a phosphate/sodium symporter that in *C. albicans* is regulated by a TRP calcium ion channels providing evidence that there is a close connection between phosphate transport and calcium metabolism (Jiang and Yang, 2018). Regulation of the core components of the PHO regulon is exerted by dephosphorylation of the master regulator Pho4 that causes its entry into the nucleus where it exerts the regulation of the PHO regulon genes or its exclusion from the nucleus. However, how the degree of phosphorylation/dephosphorylation of Pho4 does affects the biosynthesis of phosphate regulated secondary metabolites? Activation of the PHO regulon genes by the master Pho4 regulator is modulated by competitive binding of other regulatory proteins such as Pho2 and Cbf1, that recognize the same Pho4 binding sequences in the promoter of phosphate regulated genes (Zhou and O´Shea, 2011). It is likely that other still unknown transactivating or competing factors may modulate the interaction of Pho4 with its target sequences. Phosphate regulation of the biosynthesis of secondary metabolites, e.g. penicillin biosynthesis, is exerted in collaboration with carbon catabolite regulatory factors (Martín et al., 1999) but further studies on the mechanism of this concerted regulation still needs to be fully elucidated (Giots et al., 2003). Phosphate homeostasis in fungi is known to be related to the energetic and phosphorylation status of the cells that responds to the intracellular ATP *versus* AMP ratio and this is transmitted in the cells by a family of highly phosphorylated inositol pyrophosphate molecules (IP6, IP7 and higher members of the family); this provides an interesting information on how the overall phosphorylation and energetic metabolism of the cell is modulated (Secco et al., 2012; Wild et al., 2016; Azevedo and Saiardi, 2017). Regarding calcium homeostasis in fungi it is well know that calcium serves as intracellular messenger in all eukaryotic cells. The intracellular calcium concentration in fungi responds to a large variety of input signals including divalent cations, lithium, stressing pH and temperature, ethanol, caffeine, and some antifungal agents (Thewes, 2014). Filamentous fungi establish a gradient of calcium along the hyphae and this is related to the targeting of vesicles involved in secondary metabolism biosynthesis that accumulated in distal section of the hyphal tips, therefore, molecular mechanism related to calcium homeostasis and calcium gradients within hyphae are important aspects of frontier research to elucidate the secretion mechanism of bioactive metabolites. Calcium regulation of fungal metabolism is mediated by the calcium binding protein calmodulin and by its interaction with the phosphatase calcineurin. Noteworthy, calmodulin in response to calcium binding interacts directly with three different secondary metabolite biosynthesis enzymes in *B. bassiana*, particularly with the beauvericin synthetase, a non-ribosomal peptide synthetase (Kim and Sung, 2018). However, there are very few studies on this direct interaction of calmodulin with secondary metabolism biosynthesis enzymes and this field needs to be supported by additional research. A central role in the regulation of calcium homeostasis is exerted by the calcium-calmodulin-calcineurin signaling cascade in response to external stressing factors recognized by G proteins coupled receptors (GPCR). The membrane phospholipase C triggers the formation of IP3 calcium releasing factor that, therefore, increases the cytosolic concentration of this ion; the increased calcium ions bind calmodulin and triggers the calmodulin-calcineurin cascade (Figure 1). The zinc finger Crz1 (CrzA) factor in response to calcium concentration is dephosphorylated entering into the nucleus where it controls the expression of numerous genes by binding the calcineurin dependent regulatory elements. An important interaction occurs between the Crz1 factor and the phosphate metabolism as shown by the fact that Crz1 defective mutants of *S. cerevisiae* are unable to express the Pho89 transporter gene. In other word, calcium concentration in the cell controls phosphate uptake and metabolism by the Pho89 transporter in a Crz1-dependent manner. An impressive amount of fungal metabolites that include melanin and carotenoid pigments, antibiotics, antitumor agents and aflatoxins are regulated by calcium ions through CrzA (Table 2).

A third example of link between calcium and phosphate metabolism is the presence in fungi of calcisome-like vacuoles; calcisomes are membrane-bound typical calcium stores rich in polyphosphate. In these organelles calcium is bound to a polyanionic matrix of orthophosphate, pyrophosphate and polyphosphate; calcium ions may be released by alkalinization of the cells. In protozoa the cell biology of acidocalcisomes is well known; therefore, information that serves as model for these fungal calcisome-like vacuoles might be obtained by comparison of the calcium and phosphate effect on stress in these organisms. Progress in the characterization of acidocalcisomes in filamentous fungi will significantly improve our understanding of the molecular mechanism of interaction between phosphate control and calcium homeostasis.
